# Optimal scheduling in cloud healthcare system using Q-learning algorithm

**DOI:** 10.1007/s40747-022-00776-9

**Published:** 2022-06-23

**Authors:** Yafei Li, Hongfeng Wang, Na Wang, Tianhong Zhang

**Affiliations:** 1 College of Information Science and Engineering, Northeastern University, Shenyang, China; 2 Fundamental Teaching Department of Computer and Mathematics, Shenyang Normal University, Shenyang, China

**Keywords:** Cloud healthcare system, Medical resource scheduling, Markov decision model, Q-learning, ε-greedy policy

## Abstract

Cloud healthcare system (CHS) can provide the telemedicine services, which is helpful to cope with the difficulty of patients getting medical service in the traditional medical systems. However, resource scheduling in CHS has to face with a great of challenges since managing the trade-off of efficiency and quality becomes complicated due to the uncertainty of patient choice behavior. Motivated by this, a resource scheduling problem with multi-stations queueing network in CHS is studied in this paper. A Markov decision model with uncertainty is developed to optimize the match process of patients and scarce resources with the objective of minimizing the total medical costs that consist of three conflicting sub-costs, i.e., medical costs, waiting time costs and the penalty costs caused by unmuting choice behavior of patients. For solving the proposed model, a three-stage dynamic scheduling method is designed, in which an improved Q-learning algorithm is employed to achieve the optimal schedule. Numerical experimental results show that this Q-learning-based scheduling algorithm outperforms two traditional scheduling algorithms significantly, as well as the balance of the three conflicting sub-costs is kept and the service efficiency is improved.

## Introduction

With a growing high demand for medical services, the problem of overcrowding in the healthcare system becomes more prominent. It is noticeable that the high-quality medical service resources, such as the experienced specialists and the advanced medical equipment, are always concentrated in the large hospitals rather than the grass-root medical institutions. This phenomenon has intensified the problems that medical treatment is difficult and expensive for the masses [1–4]. Recently, a telemedicine medical system has newly been generated in China and termed as cloud healthcare system (CHS), which enables to accomplish the sharing of medical resources between the large hospitals and the community ones. In CHS, the IT-based medical platform can provide the telemedicine service from the specialists in the large hospitals for the patients. This new healthcare system is able to improve the service quality in the grass-root medical institutions and helpful to solve the above-mentioned problems effectively.

In recent years, a lot of researchers have begun to focus on the effect of telemedicine in the medical service systems [5–7]. Jnr et al. pointed out that telemedicine can not only effectively improve medical efficiency and reduce patient waiting time, but also help to reduce the spread of virus in the COVID-19 era [8]. Pal et al. indicated that telemedicine has great potential in increasing rural people’s access to health care in China [9]. Kumar et al. confirmed that the application of telemedicine technology not only helps to improve the efficiency of medical service while maintaining the same high service quality, but also results in improving cost and time savings for patients and healthcare providers [10]. However, it is noticeable that patient choice behaviors, i.e., appointment preference and appointment break, has to be considered when making a schedule decision in this telemedicine service based CHS. As reported in the existing literature [11–13], the telemedicine patients in CHS always break an appointment in a much larger probability than those in the traditional medical service systems.

Therefore, we will investigate the influence of patient choice behavior upon the optimal scheduling decision of CHS, which can be regarded as a special resource scheduling problem in a multi-station queueing network, in this paper. A Markov decision model with multi-types patients and multi-servers (i.e., specialists) is first developed with the objective of minimizing the total medical costs of CHS. The total medical cost consists of the online medical cost of patients, the waiting time cost, and the penalty cost of unmet patients’ choice preferences. Two decision variables are included in the model: (1) decide the matching relationship between patients and specialists, and the appointment slot according to patients’ preference; (2) make the service rules of each specialist in their available appointment slots. Then, an improved Q-learning algorithm is designed to achieve the optimal scheduling strategy according to the properties of the developed Markov model. Finally, the proposed Q-learning algorithm is verified its validity over two traditional scheduling algorithms, that is, first-come-first-service (FCFS) and priority service policy (PSP), in a series of numerical experiments.

The rest of this paper is organized as follows. In Section “Literature review”, a comprehensive review on relevant literature is given. In Section “Problem description and model”, the CHS resource scheduling problem considering patient choice behavior is modeled as a Markov model in a multi-station queueing network according to the operation characters of system. In Section “Solution method”, a three-stage dynamic scheduling method is proposed for solving the investigated problem, in which a Q-learning algorithm with an improved ε-greedy policy is designed to optimize the scheduling rule. In Section “Experimental results and analysis”, a series of numerical experiments are carried out to examine the performance of this Q-learning-based scheduling algorithm and to analyze the impact of different algorithmic parameters. In the final section, we summarize this paper and give some managerial insights drawn from the conclusions.

## Literature review

The new medical reformation in China has put forward to build the basic healthcare and complete the community-targeted health service system since 2009. Finding an effective service mode is a problem that the government and managers have been exploring since the implementation of the health care policy reform, and although some effects have been obtained, the problem of difficult and expensive access to the public is still serious. Supply-side reform of medical reported that the key problem of medical service is supply structure and quality instead of the total supply. In 2016, the policy document of “Suggestions” encouraged to build the medical environment of an Internet-based appointment triage, making cloud healthcare system (CHS) stuck out from the online medical platform.

CHS differs from the traditional medical system and other types of online consultation platforms. The existing online consultation platform is provided by a single, isolated third-party service platform without continuity between services, for instance, if the medical process is changed from online to offline, patients need to undergo some routine tests repeatedly before diagnosis [8–10]. Conversely, the medical process of patients in CHS involves multiple participants such as doctors from community hospitals and specialists from general hospitals, and the patients' visit records and examination reports can be shared among different hospitals.

We review some literature associated with our study in this paper. The comprehensive reviews and analysis about the scheduling problem in healthcare service system are provided by [14–16]. Recently, the development of CHS has brought about the widespread attention in the field of healthcare system. As a viable and significant assistant measure to the healthcare, it has achieved remarkable results in improving the service quality and medical costs of community hospitals, and contributing to the sustainability of health systems [17–19]. The provision of telemedicine services does not mean that these services will be fully utilized, more effort is needed to pay more attention for the resources management to accommodate the maximum number of service requests [20]. Some researchers have put forward that telemedicine has a good effect on the diagnosis and treatment of diabetics [21, 22]. Saghafian et al. studied a telemedicine system to decide whether to transfer online patients to the offline by the knowledge of triage nurses in community hospitals [23]. Erdogan et al. proposed the patient scheduling problem with a maximum appointment limit in the telemedicine system [24]. Buvik et al. pointed out that the key to the cost-effectiveness of telemedicine lies in the management of workload [25]. Recent studies have shown that machine learning approaches have the potential to achieve better medical results in knee joint diagnosis [26].

Patients’ choice behavior has a profound effect on the service efficiency and performance of CHS. However, the research of the patients’ preference in the telemedicine system is largely inadequate. The current research concerns traditional medical service mode. For instance, Tang et al. studied the choice behavior of patients with anxiety level in hospital [27]. Wang et al. described a framework for designing a next generation appointment system, which could dynamically learn and update patients' preferences, and used this information to improve appointment decision-making. In medical decision-making modeling, it is an important problem to evaluate patients' preferences for various health states [28]. Liu et al. examined the preferences and choices of patients in appointments of medical service to improve the patient experience by balancing speed and quality of service [29]. The capacity management problem faced by clinics was to decide which reservation requests to accept to maximize revenue. Gupta et al. established a Markov decision process model for the reservation problem, in which the patients’ choice behavior was explicitly modeled to determine the optimal control strategy and maximize the revenue of the system [30]. The study on patients’ decision behavior above has greatly improved in all respects, such as the satisfaction of patients, the resource utilization, the service quality and efficiency, but the research results are not applicable for an integrated CHS with a complicated structure. Therefore, the research in this paper fills in a gap in the aspect on the influence of patient selection behavior in CHS with multi-organizations cooperation together.

In CHS, one of the most important aspects is how to match the requirements of patients with medical resources and ensure the interests of all sites. As we all know, the customer satisfaction is important in the field of global service manufacturing. At the same time, with the continuous development of adaptive information technology, it is possible to develop a scheduling method that contains information from real-time environment to solve the complex dynamic scheduling problem in stochastic environment [31, 32]. Shiue et al. used a reinforcement learning algorithm based on multiple scheduling rules mechanism and offline learning module to maintain the knowledge base of real-time scheduling system in the dynamic environment. The scheduling results obtained by this method are more effective than other metaheuristic algorithms [33]. Wang proposed a weighted Q-learning algorithm based on dynamic greedy search to determine the optimal scheduling rule about the problem of adaptive job shop scheduling, solving the problem of blind search and improving the convergence and accuracy of the algorithm [34].

We consider a resource scheduling problem in a complex healthcare service system, which is often encountered in various fields. In this problem, CHS is composed of multiple parts, involving community hospitals, general hospitals, managers of third-party platform and patients. Referring to the works on reinforcement learning algorithm in scheduling problem, we try to apply a Q-learning algorithm to generate optimal scheduling rules in CHS.

## Problem description and model

**Fig. 1:**
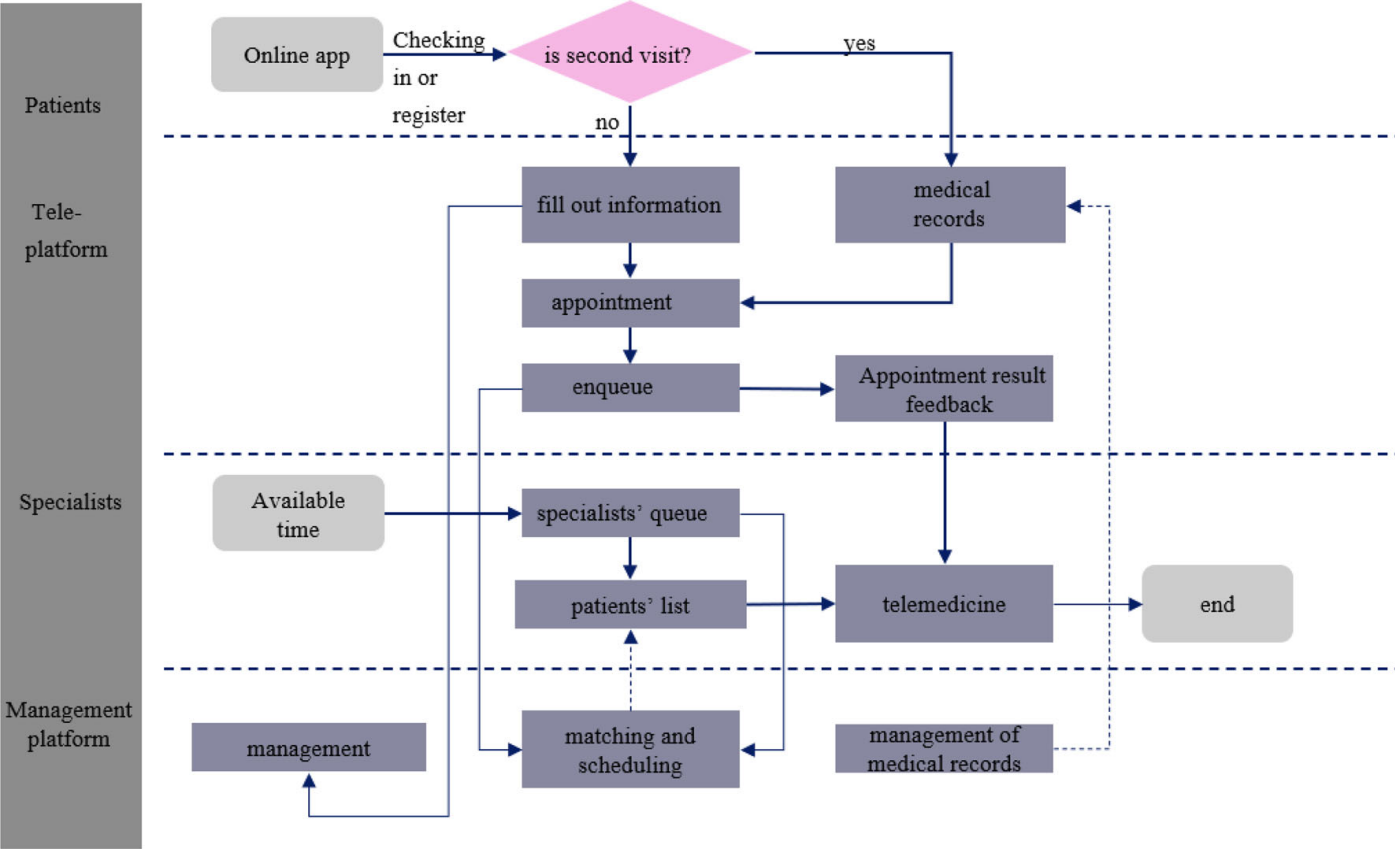
The flow chart of the CHS

As shown in Fig. 1, a simplified telemedicine service process of patients based on an actual CHS is given. In this paper, we focus on a scheduling problem regarding chronic diseases patients. There are four main parts in CHS:

(1) *Patient-side* first-time patients must fill out the detailed personal information such as name, age, illness, symptoms, and current medications using the mobile application of CHS; while returning patients can perform the function of booking register directly.

(2) *Online booking platform* the manager attaches the online shift rostered of specialists (service providers) in advance, so that patients can make medical appointment that suit their preferences about time and specialists by viewing some information about the system status such as the wait list and the available appointment slots for some specialists. Meanwhile, specialists can also early access the information about their own service queues and quickly master the patients’ illness based on the information of booking or the history diagnostic records.

(3) *Telemedicine platform* specialists provide two service modes for patients by telemedicine platform. One is by videos, audios, or graphics by the Internet technology; another is to arrange patients for referral and provide face-to-face medical service if specialists cannot provide precise performance evaluation as the limitation of network or the complexity of the illness.

(4) *Management platform* managers schedule the specialists’ resource and match it with patients by considering patients’ choice behavior to minimize the total medical costs.

Obviously, specialists are the main suppliers for medical service in the CHS, deciding how to schedule specialists and service requests is an important decision process which bears on the interests of patients and managers. Therefore, we propose a mathematical model to solve the medical resource scheduling problem with a complex network structure. The basic structural properties and some related assumptions of CHS are given below.

### Problem description

**Fig. 2:**
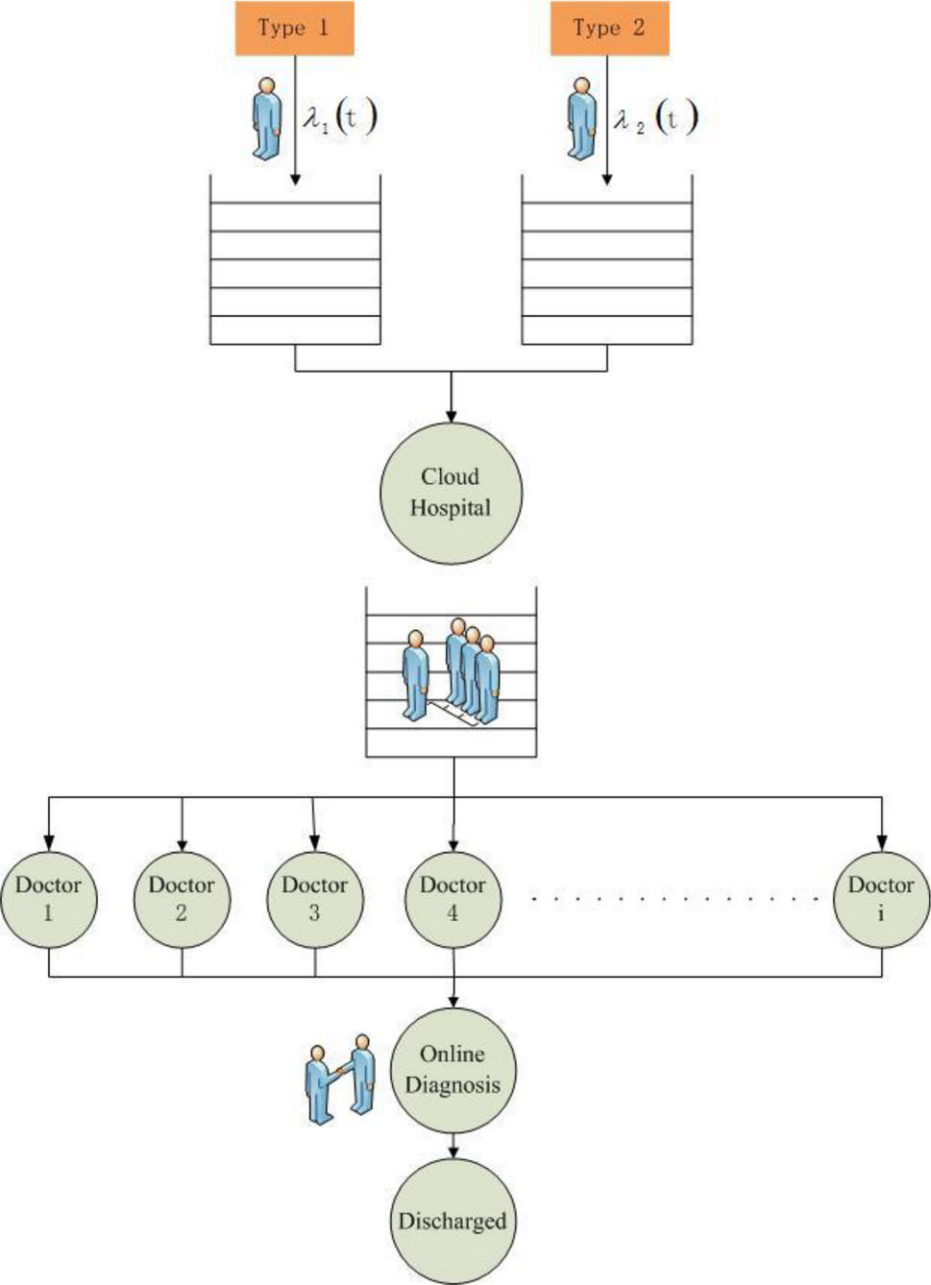
The queuing network of patients in cloud healthcare system

The queueing network of CHS is shown in Fig. 2. This paper considers two types of patients including first-time patients and returning patients, and the arrival process and service process of these patients follow different arrival rate and service rate respectively.

Due to the queueing process and the service process are complicated and flexible, we give some related assumptions of CHS before modeling as follows:

1. Appointment is performed in pre-diagnosed by telemedicine platform, and there is only one appointment request is permitted in an available slot of specialists.
2. First-time patients or returning patients have the choice of deciding specialists and slots according to their own preferences. If the selected specialist or slot is at capacity, they can wait for this specialist or consider an alternative.
3. The number of specialists and available slots are finite, and the proposed scheduling policy aims at each slot separately.
4. Patients of appointment arrival are on time.

### The impatience behavior of patient

The impatience behavior of patients has a great influence on the efficiency and the cost of the CHS, as a result, we propose a cost–benefit function to describe the acceptable queue length of patients as:

$$ {\upvarepsilon }\left( t \right) = r_{{\text{s}}} - c{*}t, $$

where $r_{{\text{s}}}$ refers to the profit of patients from services; $c$ is the waiting cost per unit time and $t$ is the expected waiting time. Patients enter the queue if and only if $\varepsilon \left( t \right) \ge 0$, which can help us to determine the number of patients arriving in unit time. We assume that the arrival process of patients follows the Poisson distribution with parameter $\lambda$, and the service times obeys Exponential distribution with parameter μ. The queue model in the CHS is a standard birth–death queue model, let $d$ be the number of specialists in the telemedicine platform, thus the stationary probability that there are $j$ patients in the CHS can be represented as follows:

$$ P_{j} = \left\{ {\begin{array}{*{20}c} {\frac{{\left( {1 - \rho } \right)\rho^{j} }}{{1 - \rho^{d + 1} }}, \rho \ne 1 0 \le j \le d} \\ {\frac{1}{d + 1},\rho = 1 0 \le j \le d} \\ \end{array} } \right.. $$

The average missed number and average queue number of patients in CHS per unit time obtained from the above formula are $\lambda_{{\text{l}}} = \lambda P_{{\text{d}}}$ and $\lambda_{i} = \lambda \left( {1 - P_{{\text{d}}} } \right)$. Furthermore, the system capacity can be calculated as:

$$ V = N_{m} + 1 = \mu \times T_{{{\text{max}}}} = \mu \times \frac{{r_{{\text{s}}} }}{c},. $$

where $\rho = \frac{\lambda }{\mu }$, $P_{{\text{d}}}$ and $N_{{\text{m}}}$ are the loss probability of patients and the maximum waiting queue of patients acceptable respectively. One of the most important concerns of managers is striking a balance between the revenue and the cost which is closely related to the service rate $\mu$. Therefore, the relationship about the revenue of CHS and the parameter $\mu$ can be presented as:

$$ f\left( \mu \right) = \lambda m\left( {1 - P_{{\text{d}}} } \right) - \omega \mu , $$

where $m$ denotes the average revenue of each patient, indicating that the value of the revenue function depends on the parameter $\mu$, thus we solve the first-derivative of $f\left( \mu \right)$ to obtain the optimal service rate $\mu$ and get the maximum value of $f\left( \mu \right)$.

$$\begin{aligned} f(\mu)^{^{\prime}} &= \lambda m\left[-\frac{{\lambda \rho^{\mu t - 1} }}{{\mu^{2} \left( {1 - \rho^{\mu t} } \right)}} - \frac{{\left( {1 - \rho } \right)\rho^{\mu t} - 1\left( {t\log \rho - \frac{\mu t - 1}{\mu }} \right)}}{{1 - \rho^{\mu t}}} \right.\\ &\quad - \left.\frac{{\left( {1 - \rho } \right)\rho^{\mu t - 1} \rho^{\mu t} \left( {t\log \rho - t} \right)}}{{\left( {1 - \rho^{\mu t} } \right)^{2}}}\right] - \omega.\end{aligned} $$

**Fig. 3:**
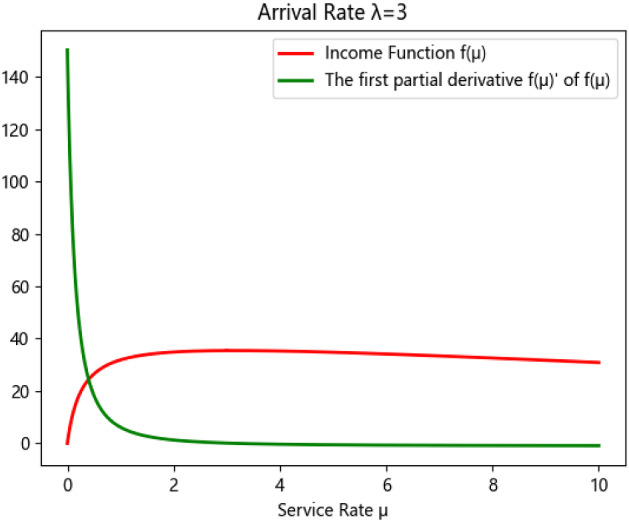
The function curve of $f\left( \mu \right)$ and $f\left( \mu \right)^{\prime }$ in unit time

Next, we design a small-scale computation case to validate the correctness of $f\left( \mu \right)$. First, we set $\lambda$ to 2, and let $m = 14$, $\omega = 1$, $r_{s} = 18$, and $c = 5$, then the relationship between the revenue function and the first-derivative of $f\left( \mu \right)$ along time is shown in Fig. 3.

By solving the equation $f\left( \mu \right)^{^{\prime}} = 0$, we can have that the optimal service rate $\mu^{*}$ is 2.511, $f\left( \mu \right)_{\max }$ = 22.700 and $V$ = 9. These results are almost same as the data provided by managers. Based on this solution, we can also determine the maximum appointment number of patients for each specialist, the service capability of the CHS for a finite time horizon, which contributes to deciding the matching problem and to ensuring the scheduling tasks performed correctly.

### Problem model

The Markov model can be established using the following notations.

The objective function can be written as follows:

$$\begin{aligned} {\mathcal{F}}^{\pi } (t) &= \mathop {\min }\limits_{t \in T} \left(\left[\mathop \sum \limits_{t = 1}^{T} \mathop \sum \limits_{i = 1}^{I} \mathop \sum \limits_{k = 1}^{K} \mathop \sum \limits_{s\left( i \right)}^{S\left( i \right)} \left( {\frac{1}{{\mu_{i,k} }}c_{i} S_{i} \left( t \right)y_{s\left( i \right)} \left( t \right) + x_{d}^{i} \left( t \right){\rm E}_{2} } \right)\right.\right.\\ &\quad \left.\left.+ \mathop \sum \limits_{t = 1}^{T} {\rm E}_{1} \left( t \right)W\left( t \right)L\left( t \right)\right]\right).\end{aligned} $$

The objective function minimizes the total costs, in which the first term represents the online medical cost of patients, the second term is the penalty cost of unmet patients’ choice preferences, and the last term represents the waiting time cost. The queueing model in CHS is a standard birth–death process, we can gain the systemic state functions by steady-state probability, which is helpful for us to solve the objective function using reinforcement learning method in the next section.

## Solution method

There are many challenges to solve the proposed model. First, patients have personal choice behavior that they can choose specialists or slots and even both. Second, there are two types of patients with different arrival rates and service rates. Third, the proposed model involves multiple conflicting objectives, how to weight each sub-objective is a difficulty mission. Reinforcement learning is a kind of method framework of learning, forecasting and decision-making that used to solve the problem that agents achieve specific goals through learning strategies in the process of interaction with the environment, when considering sequence problems, reinforcement learning has a long-term perspective and focus on the pursuit of long-term results. Therefore, in this section, we design a Q-learning algorithm based on an improved ε-greedy policy to solve the studied problem. Six key information elements of Q-learning algorithm are presented below.

1. *Environment* the environment includes the whole structure and problem description of the CHS, as shown in Section “Problem description and model”.
2. *Agent and state* the agent refers to the CHS, and the state of the agent is presented as coordinate matrix $(a,b)$, where $a$ and $b$ represent the number of first-time patients and returning patients respectively.
3. *Action* for each specialist, there are two kinds of action for choosing, one is the first-time patients, the other is the returning patients.
4. *Reward function* a reward function is designed below to ensure the accuracy and rationality of the scheduling. The agent chooses the next action and update the Q-table based on the fed back value of $\mathcal{R}\left(t\right)$ for each round.$\mathcal{R}\left(t\right)=1/ \Big(\Big[\sum_{t=1}^{T} \sum_{i=1}^{I} \sum_{k=1}^{K} \sum_{s(i)}^{S(i)} \Big(\frac{1}{{\mu }_{i,j}}{c}_{i}{S}_{i}(t){y}_{s(i)}(t)+{x}_{d}^{i}(t){\mathcal{E}}_{2}\Big) + \sum_{t=1}^{T} {\mathcal{E}}_{1}(t)W(t)L(t)\Big]\Big)$.
5. *Q-table* the structure of Q-table is presented as state $*$ action as shown in Table 2, the number of elements is $\left(M+N\right)*2D$, where $D$ is the number of specialists in the telemedicine system, and $M+N$ is the number of patients in a planning horizon

**Table 1:** Notations

Sets	
$I$	A set of specialists in the CHS,$i \in I$
$K$	A set of types of patients,$k \in K$
$S_{i}$	A set of patients served by specialist $i$, $s_{i} \in S_{i}$
$T$	A set of appointment slots every day in the planning cycle,$t \in T$
Parameters	
${\mathcal{E}}_{1} \left( t \right)$	The waiting time cost of the patients during the slot $t$
${\mathcal{E}}_{2}$	Penalty costs of unmet patients’ personal preferences
$c_{i}$	Unit medical cost of specialist $i$
$\pi$	Service rules $\pi$ of specialists
$\lambda_{k} \left( t \right)$	The arrival rate of the $k{\text{th}}$ type of patient at the beginning of the $t{\text{th}}$ time period
$\mu_{i,k}$	The service rate of specialist $i$ serving patients with type $j$
Decision variables	
$x_{d\left( i \right)} \left( t \right)$	0 if the patient served by their own designated specialist in time period $t$ and 1 otherwise
${\mathcal{Y}}_{d\left( i \right)} \left( t \right)$	1 if the patient is assigned to specialist $i$ in time period $t$ and 0 otherwise

**Table 2:** The structure of Q-table

State	Action				
	${a}_{11}$	${a}_{12}$	${a}_{21}$	$\cdots$	${a}_{D2}$
$(\mathrm{0,0})$	$Q({s}_{00},{a}_{11})$	$Q({s}_{00},{a}_{12})$	$Q({s}_{00},{a}_{21})$	$Q({s}_{00},\cdots )$	$Q({s}_{00},{a}_{D1})$
$(\mathrm{1,0})$	$Q({s}_{10},{a}_{11})$	$Q({s}_{10},{a}_{12})$	$Q({s}_{10},{a}_{21})$	$Q({s}_{10},\cdots )$	$Q({s}_{10},{a}_{D1})$
$\cdots$	$Q(\cdots ,{a}_{11})$	$Q(\cdots ,{a}_{12})$	$Q(\cdots ,{a}_{21})$	$Q(\cdots ,\cdots )$	$Q(\cdots ,{a}_{D2})$
$(M,0)$	$Q({s}_{M0},{a}_{11})$	$Q({s}_{M0},{a}_{12})$	$Q({s}_{M0},{a}_{21})$	$Q({s}_{M0},\cdots )$	$Q({s}_{M0},{a}_{D2})$
$(M,1)$	$Q({s}_{M0},{a}_{11})$	$Q({s}_{M0},{a}_{12})$	$Q({s}_{M0},{a}_{21})$	$Q({s}_{M0},\cdots )$	$Q({s}_{M1},{a}_{D2})$
$\cdots$	$Q(\cdots ,{a}_{11})$	$Q(\cdots ,{a}_{12})$	$Q(\cdots ,{a}_{21})$	$Q(\cdots ,\cdots )$	$Q(\cdots ,{a}_{D2})$
$(M,N)$	$Q({s}_{MN},{a}_{11})$	$Q({s}_{MN},{a}_{12})$	$Q({s}_{MN},{a}_{21})$	$Q({s}_{MN},\cdots )$	$Q({s}_{MN},{a}_{D2})$

We use an improved ε-greedy policy to avoid getting the local optimal solution. At a time, the agent performs an action, or exploits a new action with probability ε, or searches other actions with probability 1-ε. The expression of the ε can be designed as:

$$ \varepsilon = \frac{0.5}{{1 + e^{{\frac{{10 \times \left( {{\text{episode}} - 0.6 \times {\text{max}}\_{\text{episode}}} \right)}}{{{\text{max}}\_{\text{episodes}}}}}} }}. $$

**Fig. 4:**
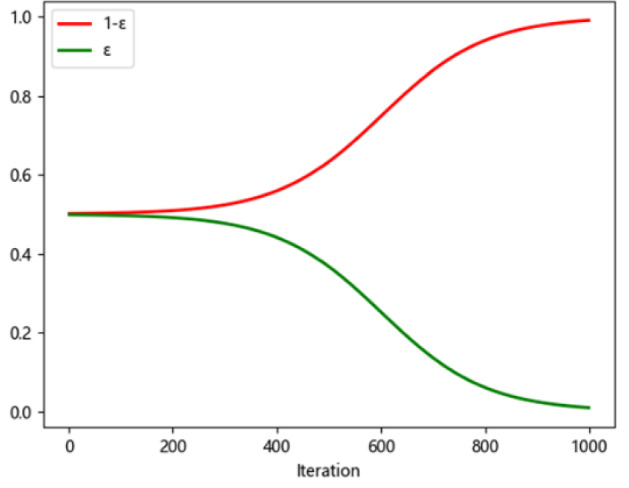
The image of *ε* and $1-\varepsilon$ varies with the number of iterations

Then, the *ε*-greedy policy is:

$$ \mu \left( {a_{t} |s_{t} } \right) = \left\{ {\begin{array}{*{20}c} {{\text{random}}\left( {A\left( {s_{t} } \right)} \right),\;{\text{rand}} > 1 - \varepsilon } \\ { a^{*} , {\text{else}}} \\ \end{array} } \right., $$

where $a^{*}$ indicates the current action $s_{t}$ when the *Q* value is maximum, and $A\left( {s_{t} } \right)$ represents a set of optional actions in $s_{t}$ state, the rand represents a sample value that obeys standard normal distribution. Figure 4 describes the changing rule about the values *ε* and 1 − *ε* with the iteration times.

**Fig. 5:**
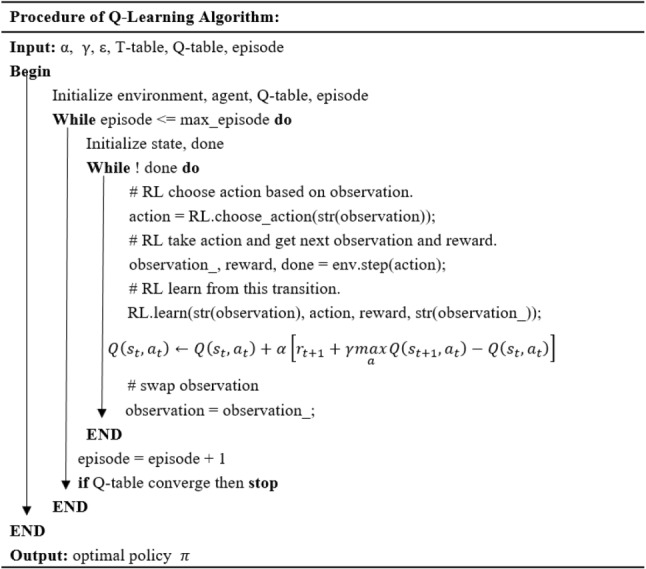
Pseudo-code of the Q-Learning algorithm

**Fig. 6:**
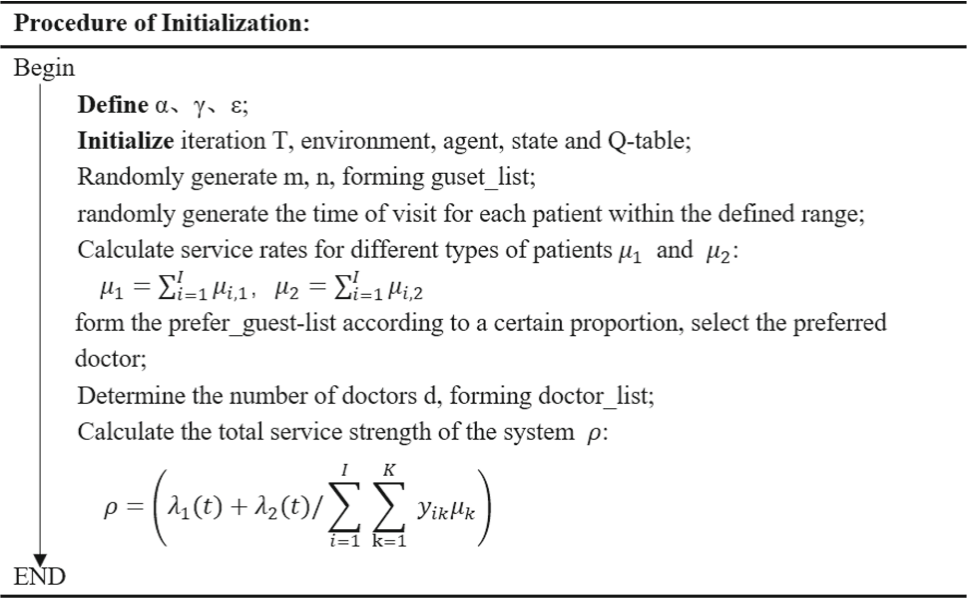
Pseudo-code of initialization procedure

Figure 4 shows that as the learning time increases the value ε gradually decrease to 0, which means that the agent has the probability of 50% to explore a new action at first, then use the knowledge that have learned from the environment to choose the best action that have learned. In this section, we present a three-stage dynamic scheduling problem using the Q-algorithm to design scheduling rules and service sequences for each specialist. At the first stage, allocating the two types of patients to each specialist under the patient’s personal preferences considered in each appointment slot. At the second stage, designing service rules for each specialist to maximize the objective function. At the third stage, deciding the optimal service sequence of patients for each specialist. The pseudo-code of the developed Q-learning algorithm is shown in Fig. 5, and the pseudo-code of the initialization process is shown in Fig. 6.

In this paper, the action refers to assigning different types of patients to different specialists, and the specific action selection process is as follows:

1. Define action space: let action space be $[{a}_{11}, {a}_{12}, {a}_{21}, {a}_{21}, \dots , {a}_{1D}, {a}_{2D}]$.
2. Initialize the system state as $(\mathrm{0,0})$, and the agent selects an action based on ε-greedy policy, i.e., the agent has the probability of ε to explore a new action, and has the probability of 1-ε to use the current action to make the next choice.
3. Judge whether the selected action meets the preference of the patient.
4. Detect whether the state_next has been stored in the state set, if not, add it to the set.
5. Calculate the *Q* value of the action performed in the current state and update the *Q* table at the same time.
6. When state = state_termanal, the selection of action ends and an episode is completed.

**Fig. 7:**
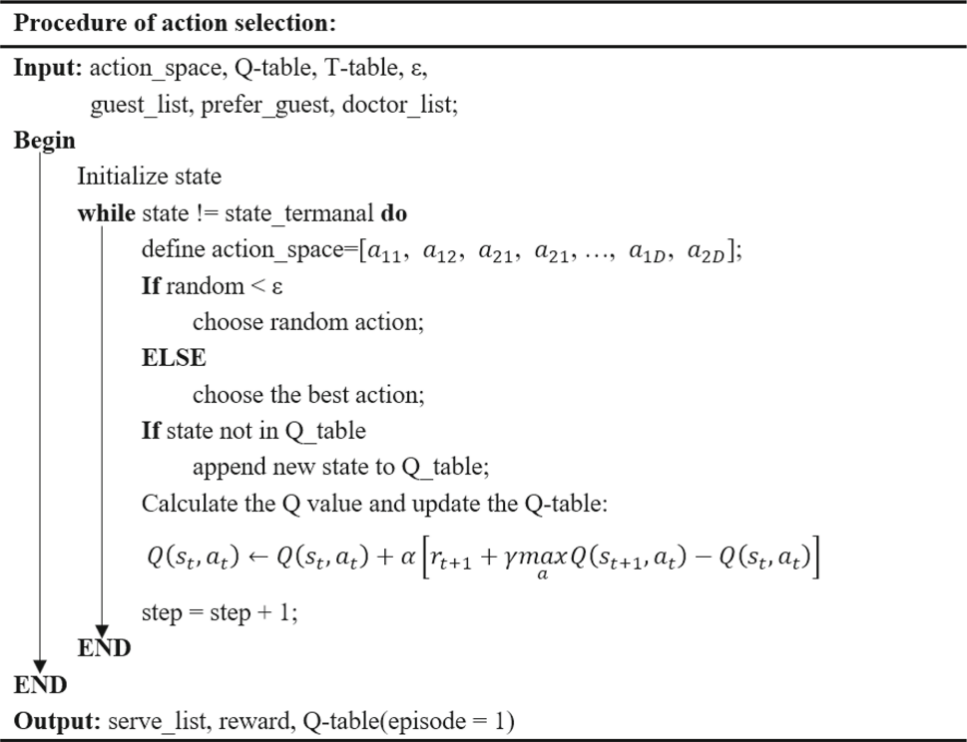
Pseudo-code of the action selection process

The pseudo-code for the action selection process is shown in Fig. 7.

**Fig. 8:**
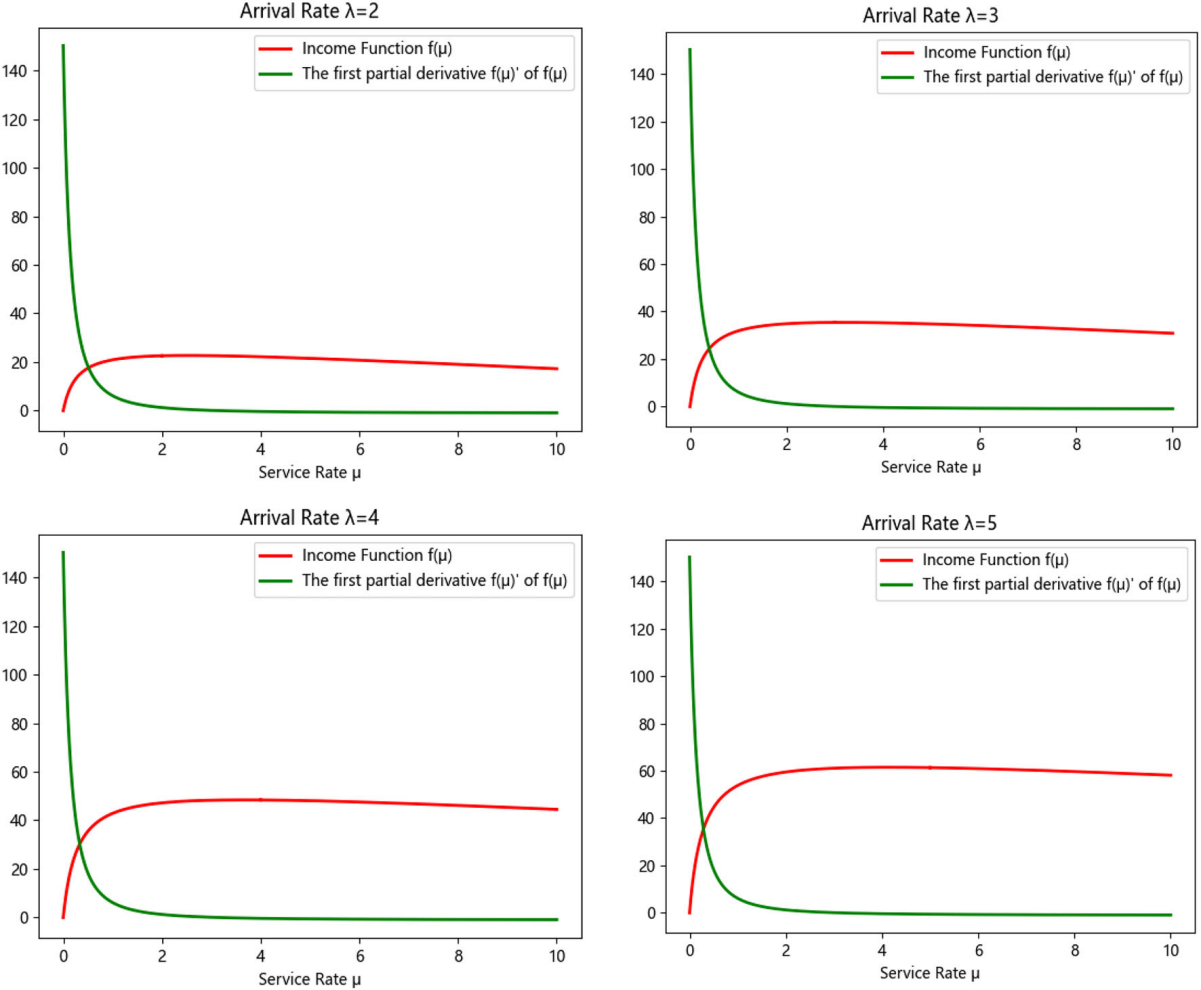
Simulation results of system capacity in different arrival rates

To calculate the reward function and update the Q-table of each iterative result based on the above pseudo-code, we can find the optimal scheduling policy when the Q-table converges. To explain the effectiveness of the proposed Q-learning algorithm more explicitly, we compare it with the traditional scheduling algorithms in the next section.

## Experimental results and analysis

Implementation of the solution methods in Section “Solution method” was implemented with Python 3.9. All experiments were run on a Lenovo Linux server with 8 GB shared RAM. To evaluate the computation performance of the developed Q-learning algorithm, we design some test cases based on six specialists and compare the Q-learning algorithm with the well-known first-come-first-service (FCFS) policy and priority service policy (PSP). PSP means that a patient must be assigned to the specialist corresponding to the patient’s choice preference, if the specialist is busy the patient has to wait until there is a usable time slot. The related parameters are set as shown in Tables 3 and 4.

**Table 3:** Parameters about specialists

Specialists	Service cost (RMB/hour)
Director A1	1000
Director A2	1000
Vice-director B1	800
Vice-director B2	800
Attending C1	600
attendings C2	600

**Table 4:** Parameters about patients

Types	Medical time	${\mathcal{E}}_{1}\left(t\right)$(RMB/minute)	${\mathcal{E}}_{2}$(RMB/person)	${\lambda }_{k}\left(t\right)$
$k=1$	15–20	15	220	1/(15–20)
$k=2$	10–15	15	380	1/(10–15)

We conduct several experiments with different parameters λ to analyze the optimal queue length for appointment of specialists in each booking slot to avoid patients waiting too long, the results are shown in Fig. 8.

**Fig. 9:**
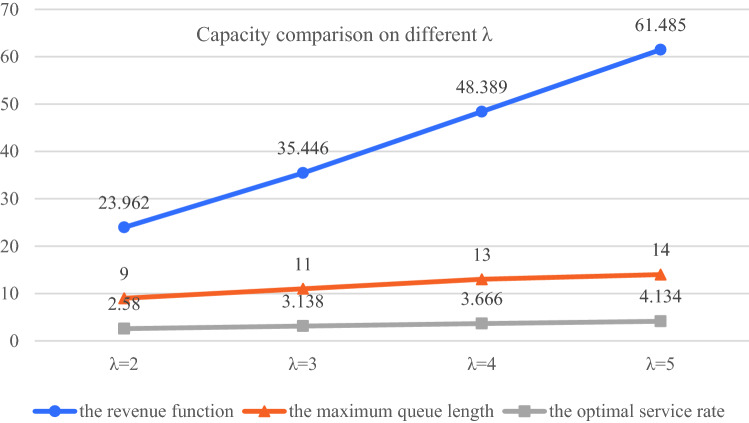
Comparison of system capacity in different arrival rates

According to the simulation results, the maximum queue length that different types of patients can accept is obtained in Fig. 9.

The experimental results show that for the above cases, the maximum queue length of specialists is 14, which is almost consistent with the data 13 provided by the manager. Based on this, we can also calculate the maximum service capacity of the system in different states.

1. FCFS scheduling rule

FCFS (first come first service) is that the scheduler always gives priority to the jobs at the top of the ready queue, and ignores any other factors. The most strength of FCFS is that it is easy to implement and fair, but it does not consider the comprehensive utilization of various resources in the system.

To describe the effectiveness of Q-learning algorithm in reducing patients' waiting time and medical costs, we build the objective function based on FCFS rules as:

$$\begin{aligned} L^{\pi } \left( t \right) &= \mathop {\min }\limits_{t \in T} \left( \left[\mathop \sum \limits_{t = 1}^{T} \mathop \sum \limits_{i = 1}^{I} \mathop \sum \limits_{k = 1}^{K} \mathop \sum \limits_{s\left( i \right)}^{S\left( i \right)} \left( {\frac{1}{{\mu_{i,k} }}c_{i} S_{i} \left( t \right)y_{s\left( i \right)} \left( t \right)} \right)\right.\right.\\ &\quad \left.\left. + \mathop \sum \limits_{t = 1}^{T} E_{1} \left( t \right)W\left( t \right)L\left( t \right) \right] \right).\end{aligned} $$

The first term of the objective function represents the medical cost of patients, and the second term is the waiting time cost. Compared with the FCFS method, the scheduling strategy of Q-learning method not only considers the patient's choice preference, but also realizes the optimal matching between patients and specialists to reduce the online visit time of patients. We schedule patients from different community hospitals in hours. The medical time of patients varies according to their conditions of illness, therefore, scheduling patients based on the rules of FCFS may lead to inefficient service due to improper matching. For example, a patient only needs 5 min under the service of specialist A, but it may take 8 min for specialist B. The specific experimental results will be discussed in detail later.

(2) PSP scheduling rule

The assignment strategy of patients is performed to the strictly according to their choice preference. While realizing the basic medical needs, considering the patient's choice behavior is very helpful to optimize the online medical configuration. For example, patients on the haodf online consultation platform (https://www.haodf.com/) can fully realize the freedom of choosing specialists and visiting time. Therefore, it is necessary to compare the scheduling method that only considers the patient's personal choice with the Q-learning method that considers the patient's choice behavior and visit time at the same time. The experimental is carried out using the parameters of FCFS.

(3) Parameters setting based on Q-Learning

The key parameters of learning algorithm are set as shown in Table 5, where the greedy coefficient ε is determined by the improved algorithm of action control in Section “Solution method”.

**Table 5:** Parameters about Q-learning algorithm

Parameters	Values
Learning rate *α*	0.1
discounted factor *γ*	0.9
iteration times *T*	500
ε	$\varepsilon =0.5/\left(1+{e}^{\frac{10\times \left(\text{ episode} - 0.6\times \mathrm{max}\_{\text{episode}}\right)}{\text{ max\_episodes}}}\right)$

### The numerical results

The work schedule of each specialist is shown in Table 6, in which specialists consist of director, vice-director and attending.

**Table 6:** Schedule of working hours for specialists

Schemes	Direc-tor A1	Direc-tor A2	Vice-director B1	Vice-director B2	Attending C1	Attending C2	Total working hours
Scheme 1	2	2	2	2	2	2	12
Scheme 2	2	2	2	3	3	3	15
Scheme 3	3	2	3	3	3	3	17
Scheme 4	4	3	4	3	3	3	20
Scheme 5	3	3	4	4	4	4	22
Scheme 6	4	4	4	4	4	4	24

**Fig. 10:**
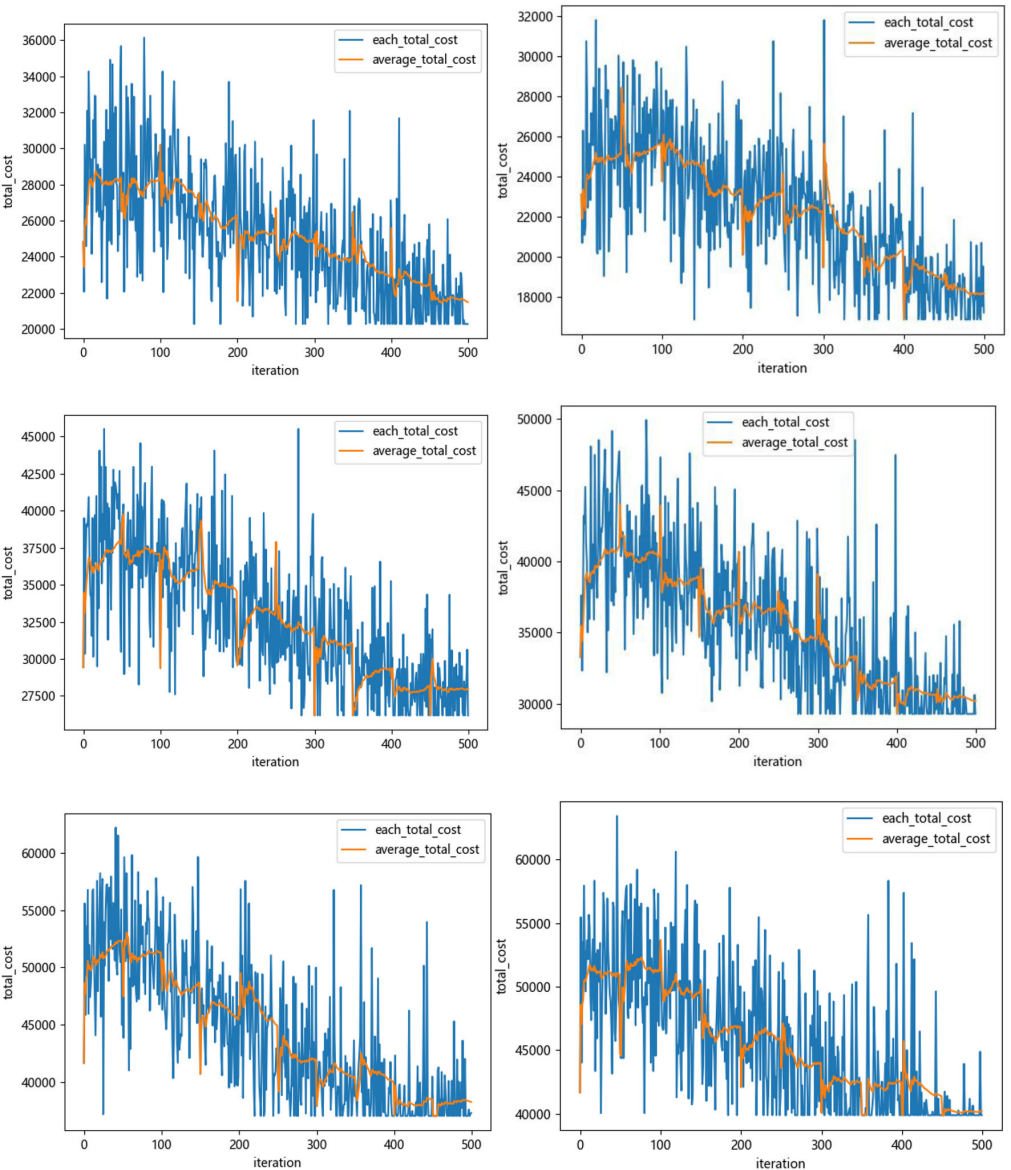
The training result of Q-Learning algorithm for Scheme 1–6 (left-to-right)

**Fig. 11:**
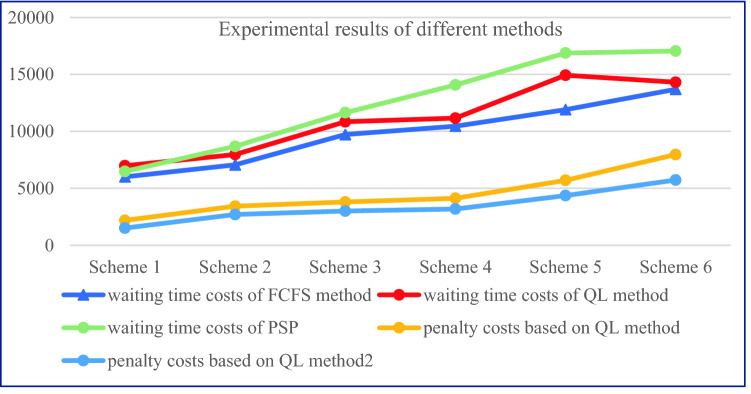
Comparison of total medical costs of different methods

Q-learning algorithm is used to learn the two rules for 500 rounds, and the training results are shown in Figs. 10 and 11, where the *X*-axis represents the number of learning rounds, and the *Y*-axis represents the total medical cost of each round of learning results.

By observing the training results based on Q-learning algorithm under six different schemes, it can be found that in the first 400 rounds of iteration process, the learning trajectory has some fluctuation due to the policies of selected action are different which is helpful to trade-off the three sub-objectives. The training results gradually converge and remain stable after 400 iterations. At the same time, we can determine the optimal scheduling strategy and corresponding service rule. The experimental results based on Q-learning algorithm, FCFS method and PSP are shown in Tables 7 and 8.

**Table 7:** The experimental comparison of Q-Learning algorithm and FCFS method

Scheme	Q-Learning				FCFS	Improvement (%)
	Medical costs	${\mathcal{E}}_{1}\left(t\right)$	${\mathcal{E}}_{2}$	Total medical costs	Total medical costs	
Scheme 1	8374	14,306	4728	19,871	27,780	21.9
Scheme 2	9606	13,929	4360	27,895	36,571	31.1
Scheme 3	12,338	11,157	3183	26,678	36,269	26.4
Scheme 4	14,937	10,841	3002	28,782	40,082	28.2
Scheme 5	18,760	7970	2694	29,424	48,043	38.7
Scheme 6	22,852	7085	1506	31,443	45,323	30.6

**Table 8:** The experimental comparison of Q-Learning algorithm and PSP

	Q-Learning	PSP	Improvement (%)
Scheme	Total medical costs	Total medical costs	
Scheme 1	19,871	29,014	31.5
Scheme 2	27,895	39,905	30.1
Scheme 3	26,678	41,427	35.6
Scheme 4	28,782	42,809	32.8
Scheme 5	29,424	44,105	33.3
Scheme 6	31,443	44,682	29.6

From Tables 7 and 8 we can see that the Q-learning method has a significant improvement in three sub-costs which makes the total medical costs can be saved up to 38.7%, this means that the Q-learning method can effective way to solve the scheduling problem in complex CHS. The scheduling policy of Q-learning method is not strictly following the patients’ choice preference compared with the PSP instead to balance the preference and waiting time of patients. The PSP policy allocates patients according to their personal choice, which may lead to the idle of other specialists, thus increasing the waiting time cost of patients.

**Fig. 12:**
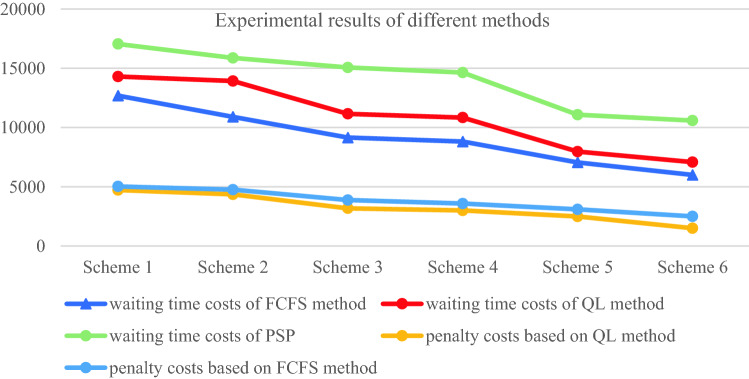
The results comparison between Q-Learning algorithm, FCFS method and PSP

**Fig. 13:**
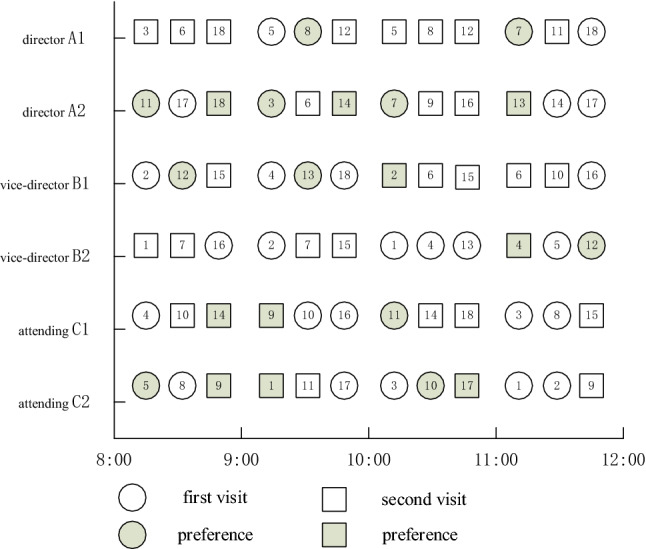
Scheduling Gantt diagram based on FCFS method

**Fig. 14:**
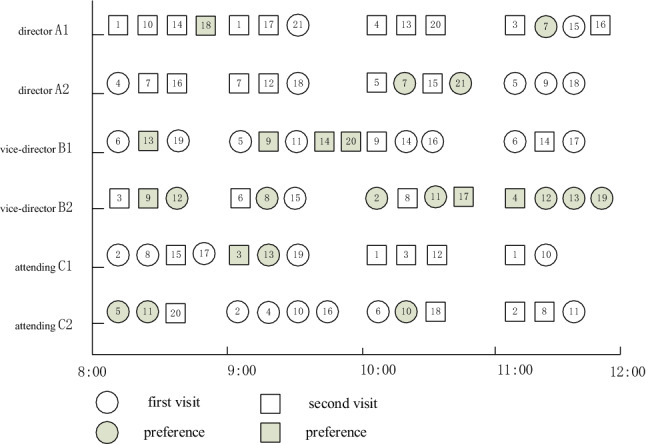
Scheduling Gantt diagram based on PSP

**Fig. 15:**
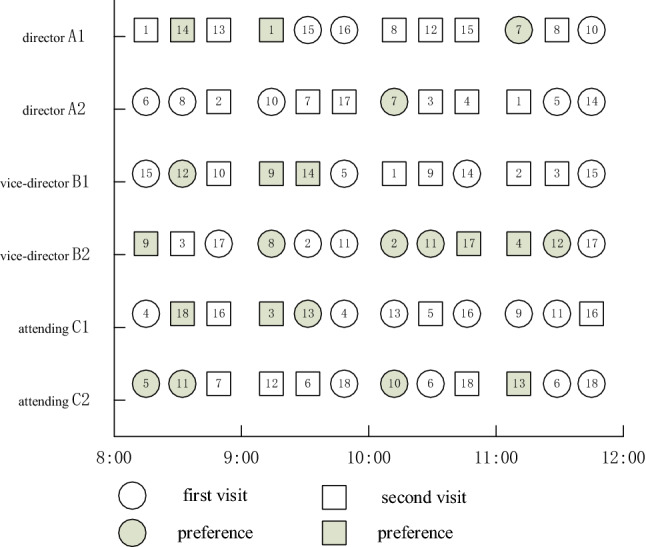
Scheduling Gantt diagram based on Q-learning algorithms

Figures 11 and 12 show that the Q-learning algorithm is better than FCFS method which ignores the patients’ choice preference. The waiting time cost based on the FCFC method is smallest because the patients are served according to their arrival sequence, so the penalty cost is higher. The PSP is strictly following the patient’s choice preference no matter how the queue is long which makes the other resource cannot be fully utilized and leads to a higher waiting time cost. In Fig. 12, the penalty cost and waiting time cost gradually decrease because the waiting time of patients decreases and patients have more opportunities to be assigned to the specialists they choose. However, the decreased slightly when the working hours of specialists increase to some extent when the number of patients is fixed. Above experiments can provide the managers with effective decision-making references that to balance the number of specialists and the medical costs. Therefore, the Q-learning method is the most effective way to balance the multiple conflicting sub-objective function. The scheduling results based these three methods are shown as Figs. 13, 14 and 15 respectively.

Figure 15 is the scheduling Gantt chart of scheme 6 after 500 rounds of learning. It not only reflects the matching relationship between specialists and patients, but also expresses the personal choice preference of patients. In each appointment slot, we give priority to patients who have made an appointment in the previous slot to reduce the waiting time of patients. The right only shows the scheduling results of some patients, and the numbers in this figure are marked according to the arrival order of patients. It is obvious that the matching between doctors and patients and the scheduling results of patients are quite different from the results in Fig. 13. This is because FCFS rules completely ignore the patient's personal choice preference, patient’s type and specialist's service rate and other factors, and strictly schedule according to the patient's appointment time. Figure 14 is the scheduling result of PSP, which follows strictly patient’s choice preference, which leads to high waiting time cost and resource waste. For example, the queue of specialist B_1_ is longer in the beginning time slot 9:00–10:00 than that of other specialists in the beginning time slot 9:00–10:00. The above experiments indicate the effectiveness of the scheduling results of Q-learning algorithms than the FCFS method and PSP.

The experimental results can provide decision support for managers, and help them to define the optimal matching relationship and service rules of patients with personal choice preference in the queuing system with multi-types of patients and multi-server, this is also having the instructive significance for other organizations with scarce resources.

### The improved Q-learning algorithm

We improve the Q-learning algorithm by introducing the ε (episode) function to make the agent has the equal chance to select an action from the action sets. To verify the validity of the improved algorithm of action selection policy, we conduct some comparative test follows the parameters value with Table 5, and the other parameters shown in Table 9 about the improved algorithm:

**Table 9:** Experimental parameters of improved Q-Learning algorithm

parameters	values
Learning rate α	0.1
discounted factor γ	0.9
iteration times T	500
ε	$\upvarepsilon =0.5/\left(1+{e}^{\frac{10\times \left(\text{ episode} - 0.6\times \mathrm{max}\_{\text{episode}}\right)}{\text{ max\_episodes}}}\right)$

**Fig. 16:**
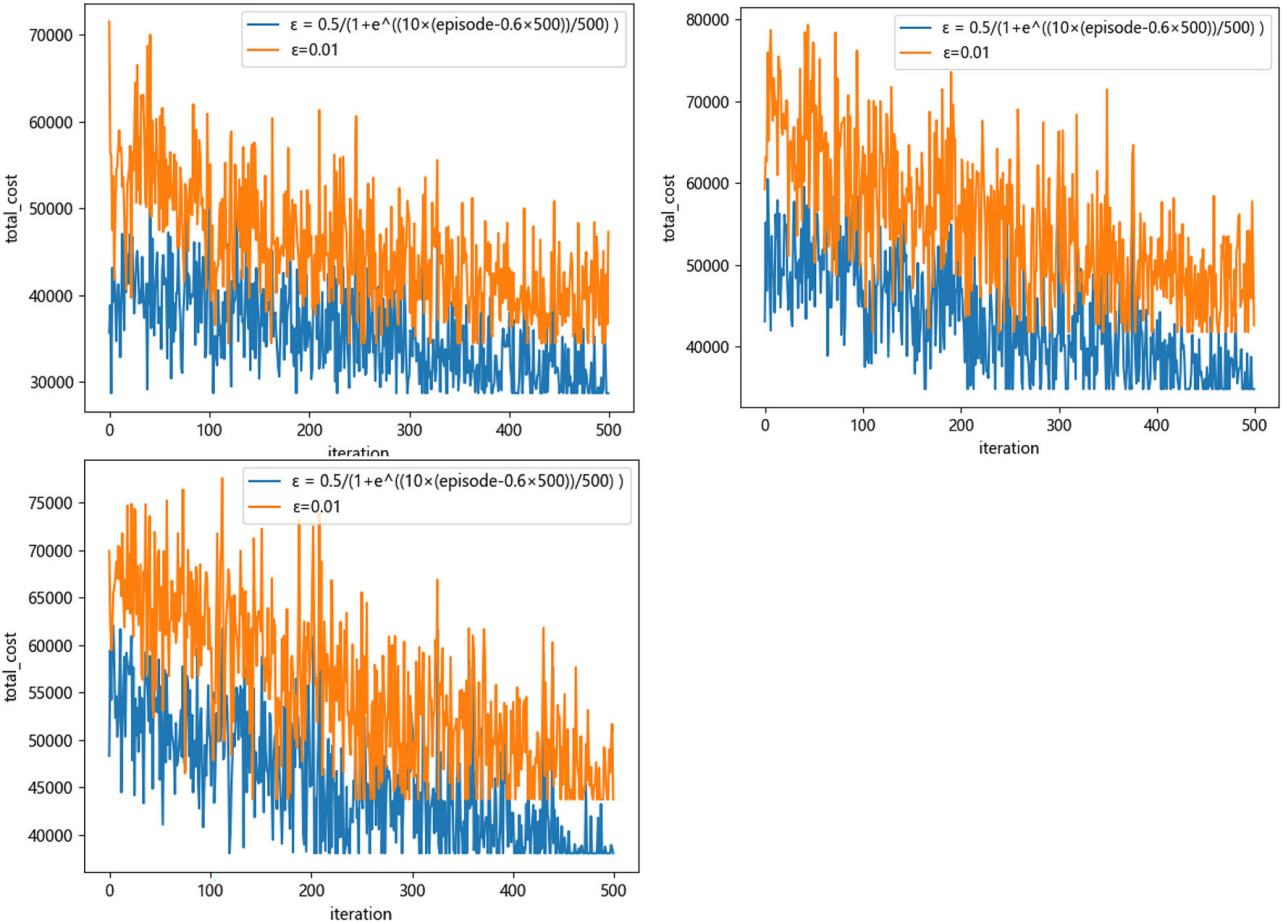
The function values for Schemes 4–6

The experiments based the scheme 4–6 of Table 6 are performed, we modify the selection policy of action $\upvarepsilon$ that assigned the value $\varepsilon =0.5/\left(1+{e}^{\frac{10\times \left(\mathrm{episode}-0.6\times 500\right)}{500}}\right)$ and compared the value of medical costs with the $\varepsilon =0.1$. The results obtained from different selection policy are shown in Fig. 16.

The simulation results from the several groups of experiments indicate that the improved Q-learning method has higher efficiency and faster convergence speed than the unimproved, as well as makes the performance rises at least 16.9%. Take scheme 6 as an example, the reward value (rounded) gradually increases of training result for each state in Q-table with the change of color from blue to red which are shown in Table 10:

**Table 10:**
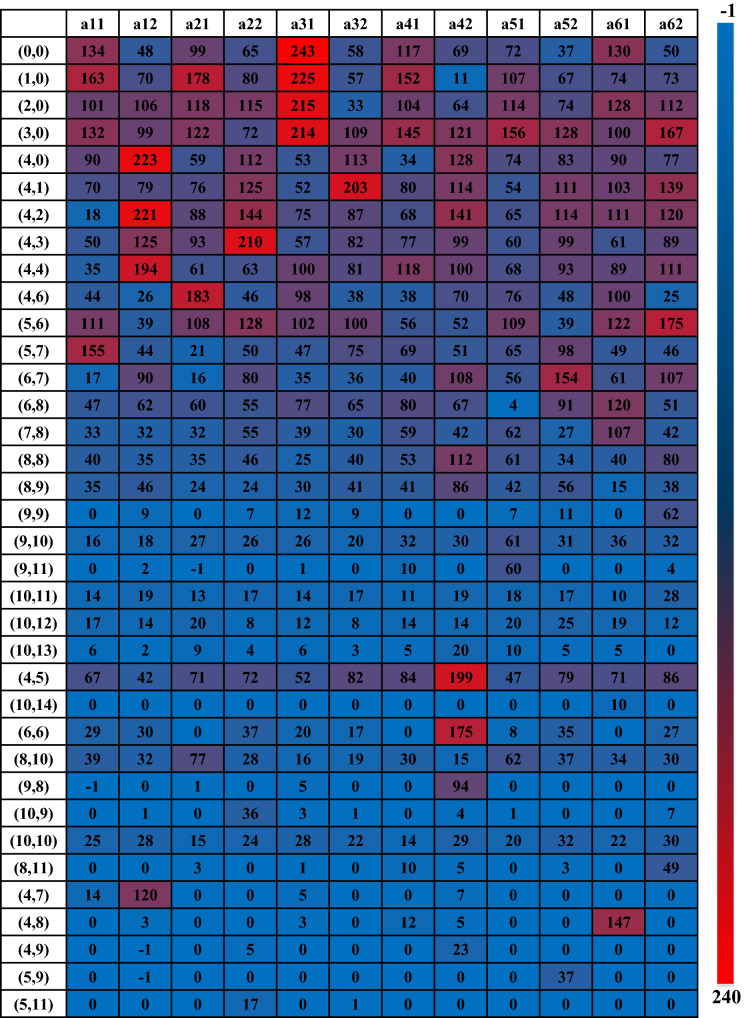
Q-table results of improved Q-learning algorithm

Our experimental results show that compared with the traditional FCFS rules and PSP, the improved Q-learning algorithm proposed in this paper makes the performance increased by at least 16.9% in solving the scheduling problem of complex CHS. We present a state space with "urgency" and a return function with "delay penalty" which take the total medical cost of the system as the performance index, and design an action selection strategy with "more random in the early stage" and "more accurate in the later stage" in the whole learning process, to give an optimal scheme and some scheduling rules at each appointment slot.

## Conclusions

In this paper, we investigate an optimal scheduling problem in cloud healthcare system (CHS) that is presented as a multi-station queueing network. A Markov model is developed considering that the patients in CHS always have the uncertain choice preference on the medical specialists and appointment slots. To achieve the optimal CHS scheduling decision, an improved Q-learning algorithm is proposed and verified the validity by conducting a series of numerical experiments. The experimental results that the proposed Q-learning algorithm has the better performance than two traditional scheduling algorithms in terms of the acceptable queue length and the service capacity limitation. According to the analytical insights, the developed model and the proposed algorithm in this paper can provide a good tool for the managers in improving the medical service efficiency in CHS.

There are some limitations to our study that present opportunities for future research. In our proposed Q-learning algorithm, Q-table would become very large with the growth scale of the investigated problem and hard to converge. Though the limitation of service capacity had been given in the numerical experiments, it is an important future work to hybridize intelligent optimization algorithms, such as genetic algorithm, Tabu search and etc., into the framework of Q-learning algorithm for solving the more large-scale scheduling problem. Moreover, the basic assumptions are simplified in the investigated system. Many factors, i.e., the actual medical process of CHS, the patients’ choice behaviors and so on, would be more complicated in the real-world scenario, which is also to explore future in the future.
